# Factors associated with wheezing in Indigenous children and adolescents: A systematic review of the global literature

**DOI:** 10.1371/journal.pone.0345711

**Published:** 2026-03-27

**Authors:** Marcia Corrêa de Castro, Daniella Campelo Batalha Cox Moore, Karla Gonçalves Camacho, Saint Clair dos Santos Gomes, Margarida dos Santos Salú, Zina Maria Almeida de Azevedo, Sandra Lisboa, Andrey Moreira Cardoso

**Affiliations:** 1 Education Department, Fernandes Figueira National Institute of Women, Children, and Adolescent Health-FIOCRUZ, Rio de Janeiro, Brazil; 2 Pediatrics Department, Fernandes Figueira National Institute of Women, Children, and Adolescent Health-FIOCRUZ, Rio de Janeiro, Brazil; 3 Internal Medicine Department, School of Medicine, Fluminense Federal University, Rio de Janeiro, Brazil; 4 Perinatal Center, Rio de Janeiro State University-UERJ, Rio de Janeiro, Brazil; 5 Clinical Research Department, Fernandes Figueira National Institute of Women, Children, and Adolescent Health-FIOCRUZ, Rio de Janeiro, Brazil; 6 Pediatrics Department, D’Or Institute for Research and Education-IDOR, Rio de Janeiro, Brazil; 7 Samuel Pessoa Department of Endemic Diseases, Sergio Arouca National School of Public Health-FIOCRUZ, Rio de Janeiro, Brazil; Hokkaido University: Hokkaido Daigaku, JAPAN

## Abstract

**Background:**

Wheezing episodes are common in childhood and can occur due to different health conditions. Globally, Indigenous children are particularly affected, with a high burden of respiratory diseases related to socioeconomic inequalities, demographic characteristics, and exposure to environmental risks.

**Objective:**

We summarize the knowledge available in the global literature on the determinants of wheezing in Indigenous children and adolescents and develop a theoretical model for analyzing them in Indigenous Brazilian children.

**Methods:**

Systematic review conducted under the PRISMA 2020 criteria for studies, registered in PROSPERO (CRD42023395661). The Medline, Scopus, Web of Science, and LILACS databases were searched until September 2024. The inclusion criterion was analytical observational studies that showed risk factors for wheezing in children and adolescents (0–19 years). Studies that did not show specific results in the age group of interest, even if they included children and adolescents in their samples, book chapters, conference annals, or outcomes associated with chronic lung disease complications were excluded. Quality assessment was performed using the Newcastle-Ottawa Scale.

**Results:**

Seventeen of the 263 analyzed studies were included in this systematic review, with a total of 139,783 participants. The most common design was cross-sectional (76.5%); most studies were conducted in North America (76.5%). Asthma was the most common type of wheezing episodes (88.2%). Male patients showed a more significant association with wheezing outcomes. Wheezing, when combined with asthma, was directly associated with age and inversely associated with age when accompanied by cold.

**Conclusion:**

We identified three main factors for wheezing in Indigenous children and adolescents: Environmental (tobacco smoke, indoor pollution, and housing), socioeconomic (income, healthcare, and residence), and biological/clinical (sex, birth weight, infections, and allergies). We propose a model to analyze these factors in Indigenous Brazilian children. Differences in definitions, reliance on numerous cross-sectional studies, and focus on North America limit the generalizability of the results. Standardized methods and studies in new regions, particularly Brazil, are necessary for testing and refine this model.

## Introduction

Wheezing is the sound of airflow passing through the airways when affected by any condition that leads to narrowing or obstruction hindering the respiratory cycle [1]. It is a clinical manifestation frequently associated with inflammatory processes of the airways, particularly the bronchioles, and is a common childhood disease in the first two years of life [1–8]. The respiratory syncytial virus (RSV) is the etiological agent most commonly associated with wheezing in bronchiolitis. However, other factors have been associated with this manifestation, such as maternal tobacco use during pregnancy [9–11], perinatal or neonatal complications that affect the development and maturation of the infant’s respiratory system, adverse intra-household environmental conditions during the postnatal period [7,12] and a variety of health conditions, such as the newborn’s transient tachypnea, asthma, gastroesophageal reflux disease, inborn errors of immunity, foreign body in the airway, vascular ring, bronchopulmonary dysplasia, cystic fibrosis, tracheoesophageal fistulas, primary ciliary dyskinesia, tumors, bronchiectasis, and congenital heart disease [5,13–15].

Isolated or recurrent wheezing episodes, occurring three or more times during the first year of life, can negatively affect the health and quality of life of children and their caregivers [11,16,17]. Routine visits to emergency services, hospitalizations with respiratory support, and prolonged use of medications, combined with adverse socioeconomic conditions, affect child’s overall development.

In the context of the first wheezing episodes and their repercussions in childhood, a multicenter longitudinal study on allergies conducted in children from birth to 13 years of age in Germany found heterogeneous manifestations of wheezing episodes in childhood, highlighting the importance of monitoring child for early diagnosis based on age at the first episode. The incidence of the first episode was the highest in the first year of life (18%) and decreased over the subsequent two years [18]. In South Brazil, 62.7% of low-income children had wheezing episodes in the first year of life, starting at five months of age, resulting in high frequencies of emergency room visits (58.2%) and hospitalization (16%) [17]. Thus, identifying children with a predisposition to wheezing from birth or the first episode and providing adequate monitoring can contribute to early identification of wheezing patterns and their determinants, guide clinical management and prevention, and ultimately improve child health.

In developed countries such as the United States, Canada, New Zealand, and Australia, the burden of respiratory morbidity has been higher among Indigenous children when compared with non-Indigenous children [19–25]. A systematic review conducted in Australia found higher rates of RSV bronchiolitis, hospitalization, and ventilatory support in Indigenous infants aged 0–6 months than in non-Indigenous infants; these inequities were associated with inequalities in access to health services, housing conditions, and tobacco smoke exposure [12]. In Australia, a higher prevalence of severe wheezing episodes was found among Indigenous people aged 6–8 years (45.9%) than among non-Indigenous people of the same age group (31.5%) [26]. Despite the disparities in the occurrence of wheezing and its complications between Indigenous and non-Indigenous people and the diverse life contexts to which these population segments are subjected, a brief survey of systematic review and meta-analysis studies on “wheezing or respiratory sounds” and “associated factors or risk factors” in children aged 0–18 years conducted in October 2024 in the MEDLINE bibliographic database for the last 10 years identified 20 publications that included the term “wheezing” in the title or abstract, but none of them included Indigenous children.

In Brazil, Indigenous people also have a high burden of respiratory diseases compared with the general population, particularly children [27–29]. Studies on the respiratory health of children in Guarani, one of the largest ethnic groups in the country, have shown the relevance of wheezing in Indigenous childhood morbidity profiles. For instance, based on hospitalization data of Guarani children under five who were prospectively followed for three years to conduct a case-control study on factors associated with the incidence of hospitalization due to acute respiratory infection of the lower respiratory tract, a prevalence of wheezing of 64.7% was identified in hospitalized children under one year of age, with symptoms concentrated in the autumn in the Southern Hemisphere (April–June) (64.2%). Wheezing was the main comorbidity associated with hospitalization [30]. There was a higher proportion of wheezing among Indigenous children hospitalized due to acute respiratory infections of a presumed viral etiology (40.8%) [31]. The relevance of acute respiratory diseases and wheezing among Indigenous Guarani children prompted the creation of the first cohort of Indigenous births in Brazil, the Guarani Birth Cohort, which aimed to describe the wheezing profile in the first year of life and identify factors associated with the incidence of wheezing in the cohort. This first systematic review of world literature on factors associated with wheezing in Indigenous children and adolescents aimed to summarize the current knowledge on the subject and support the development of a theoretical model for analyzing the determinants of wheezing in Indigenous Guarani children in Brazil.

## Materials and methods

We followed the PRISMA (Preferred Reporting Items for Systematic Reviews and Meta-Analyses) guidelines, which are used for reporting systematic reviews and meta-analyses. We adhered to the guidelines of the Joanna Briggs Institute (JBI). These guidelines offer methods for conducting systematic reviews. The study was registered with PROSPERO (CRD42023395661), an international registry for systematic review protocols, on July 7, 2023. The initial search was conducted in March 2023 and updated in September 2024. We found no new studies beyond the initial searches in MEDLINE*, Web of Science, Scopus, and LILACS (Latin American and Caribbean Health Sciences Literature).* Readers can replicate our search strategy across relevant databases using the search terms and strings listed in S1 Supplement.

Reporting Compliance: We used the PRISMA 2020 checklist. This tool outlines the steps for reporting systematic reviews. This ensured reproducibility and transparency during the review. We have defined the responsibilities of the reviewers. We provided the full search strings.

The search terms were organized using a scientific library. We used *Medical Subject Headings (MeSH),* a standardized keyword system for indexing biomedical information; *Health Sciences Descriptors (DeCS),* a similar standardized term system used in *Latin America and the Caribbean,* as well as keywords from related studies, either alone or in combination, to meet the inclusion criteria and maximize sensitivity. We searched titles, abstracts, and keywords in English and Portuguese, adapting the terms to suit the specific requirements of each database. For instance: ((wheezing OR bronchiolitis OR bronchospasm OR asthma) AND ((“indigenous infants”) OR (“native children”) OR (“indigenous children”) OR (“indigenous population”) OR (“Indians central American”) OR (“North American Indians”) OR (“South American Indians”) OR (“health of indigenous peoples”) OR (“Australian Aborigines”) OR (“First Nations”))) AND (pediatric OR infants OR children) AND (risk factors OR associated factors OR etiology OR causes OR protective factors). Database-specific search terms are provided in S1 Supplement.

### Study eligibility and selection stages

Bibliographic references were managed by the Covidence software (https://www.covidence.org). The selection resulting from the database search was conducted at different stages within the platform and under the criteria established for this research: 1) import of references in RIS or PubMed XML formats; 2) identification and exclusion of duplicate articles; 3) evaluation of titles and abstracts; 4) evaluation of full-text articles; 5) storage of articles in portable document format (PDF); 6) data extraction; and 7) quality analysis. During the title and abstract reading and full-text article analysis stages, the eligibility criteria were evaluated considering the previously defined inclusion and exclusion criteria. The inclusion criteria were studies involving Indigenous children or adolescents aged 0–19 that evaluated factors associated with wheezing or other outcomes of wheezing, such as bronchiolitis and asthma, and were restricted to analytical studies with a comparison group. There were no limitations regarding the language, data collection period, publication, or study design. We excluded studies without separate results for Indigenous children or adolescents, editorials, letters, book chapters, reviews or meta-analyses, conference abstracts, and studies in which wheezing was related to chronic lung disease complications, such as bronchiectasis, as well as unpublished articles. Two researchers (MCC and KGC) independently analyzed the texts selected at each stage, with a third researcher (DCBCM) serving as an arbitrator in cases of reaching no consensus.

### Data extraction

Regarding data extraction, a form adapted from Covidence software was used, covering the following topics: general information, characteristics of the included studies, risk factors (sociodemographic, environmental, healthcare, biological, infectious, etc.), statistical analysis, and conclusions.

### Assessment of methodological quality and risk of bias

Quality and risk of bias assessments were performed using the Newcastle-Ottawa Scale (NOS) [32] for case-control and cross-sectional studies. The NOS is a methodological quality rating tool for non-randomized studies that assigns points based on the selection, comparability, and assessment of outcome/exposure. According to the scale scores, the studies were categorized as having high quality and low risk of bias (≥8 points), moderate quality and moderate risk of bias (6–7 points), and low quality and high risk of bias (<6 points). The domains and scores are presented in the supplementary material (S2 Supplement).

### Summary of results

We performed a descriptive synthesis of study characteristics and results included in this review. A descriptive synthesis summarizes findings narratively rather than statistically. The synthesis detailed the lead author, year of publication, country, associated factors, outcomes, and ratio-type effect measures (odds ratio [OR] or relative risk [RR]) along with their respective confidence intervals (CIs), as presented in S1 Table in S6 Supplement. ORs and RRs are statistical measures that indicate the strength of the association between risk factors and outcomes, whereas CIs show the reliability of these estimates.

The results were grouped by continent and study quality to facilitate comparisons and reveal geographical patterns in the study outcomes. The subgroup themes focused on environmental factors, healthcare access, and socio-demographic variables, enabling a comprehensive comparative analysis of the unique challenges and determinants of wheezing across diverse global contexts.

## Results

### Evidence source

**Fig 1:**
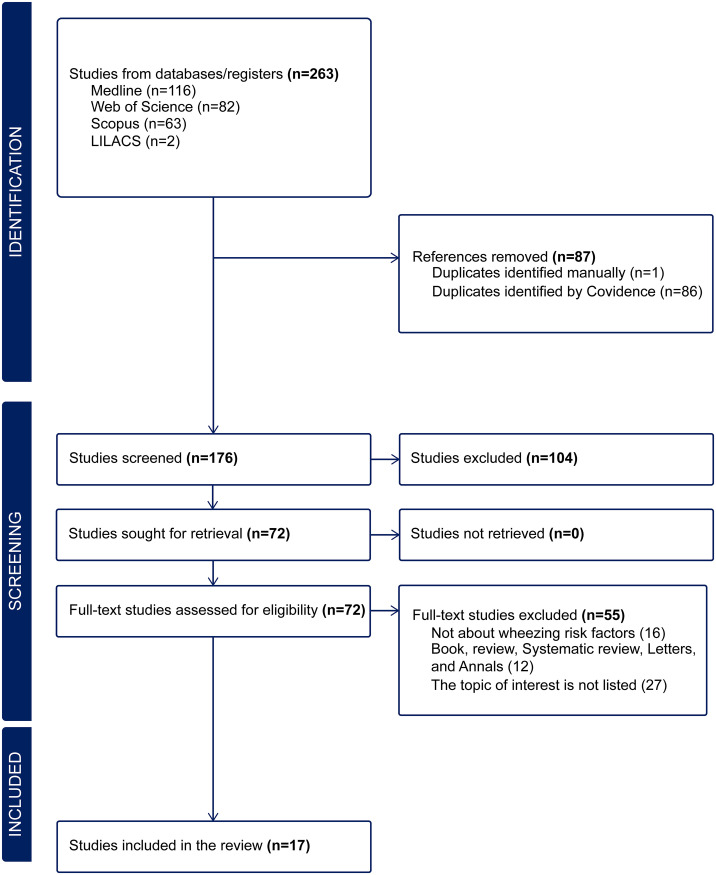
PRISMA flowchart of studies included in the systematic review of global literature.

**Fig 2:**
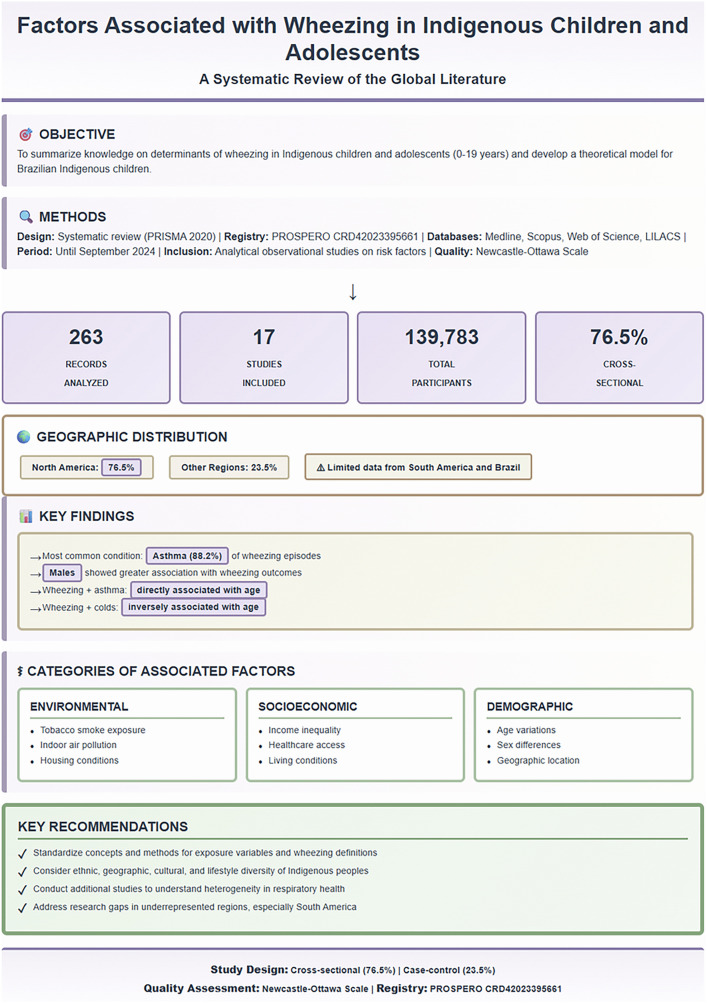
Visual abstract summarizing the systematic review on factors associated with wheezing in Indigenous children and adolescents. The figure shows: (A) study objective and inclusion criteria; (B) systematic search methodology following the PRISMA 2020 guidelines across four databases (Medline, Scopus, Web of Science, LILACS); (C) quantitative results revealing 17 included studies from 263 initial records, encompassing 139,783 participants, with predominance of cross-sectional designs (76.5%); (D) geographic distribution demonstrating concentration in North America (76.5%; Canada 41.2%, USA 35.3%) with limited representation from South America (11.8%), Central America (5.9%), and Oceania (5.9%), and absence of studies from Asia and Africa; (E) key clinical findings including asthma as the most common condition (88.2%), male predominance, and age-related associations; (F) three main categories of identified associated factors: environmental (tobacco smoke, indoor pollution, and housing conditions), socioeconomic (income, healthcare access, and living conditions), and demographic (age, sex, and geographic location); and (G) key recommendations including standardization of methods, consideration of ethnic and cultural diversity, and addressing research gaps in underrepresented regions. Quality assessment performed using the Newcastle-Ottawa Scale. PROSPERO registration: CRD42023395661.

In total, 263 publications were identified. After excluding 87 duplicate articles, 176 articles were screened after reviewing their titles and abstracts. During the screening, 104 studies that did not meet the established inclusion criteria were excluded. The remaining 72 articles were subjected to full-text review. At this stage, 55 articles were excluded, leaving 17 studies for further analysis (Fig 1). Fig 2 provides a comprehensive visual overview of the systematic review process, geographic distribution of studies, key findings, and main categories of factors identified. This figure synthesizes the results from 17 studies including 139,783 Indigenous children and adolescents across North America (76.5%), South America (11.8%), Central America (5.9%), and Oceania (5.9%), highlighting the predominance of cross-sectional designs (76.5%) and the need for research in underrepresented regions.

### Individual characteristics and results of evidence sources

**Fig 3:**
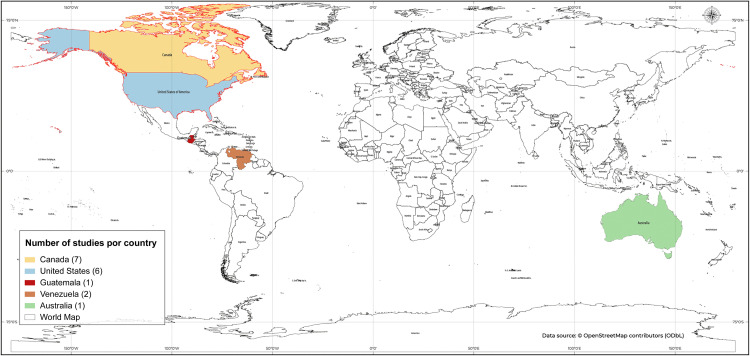
Geographic distribution of studies included in the systematic review. World map showing 17 studies across 5 countries in the Americas and Oceania. Countries were color-coded according to the number of included studies: Canada (7, yellow), the United States (6, blue), Venezuela (2, orange), Guatemala (1, red), and Australia (1, green). Country boundaries were obtained from the OpenStreetMap contributors (www.openstreetmap.org) using the Open Database License (http://opendatacommons.org/licenses/odbl/1.0/).

The years of publication of the studies extended from 1995 to 2022; however, with the exception of one study published in 1995, the others were published from 2004 onwards. The studies were conducted predominantly in North American countries (13/17, 76.5%), followed by South America (2/17, 11.8%) and Central America and Oceania, both with only one study (1/17, 5.9%) (Fig 3). All studies were published in English.

The most common study design was cross-sectional (13/17, 76.5%), followed by case-control (4/17, 23.5%). No other study design was identified. Four studies (23.5%) investigated more than one ethnic group, ranging between two (one study) and four (two studies) ethnic groups.

Asthma was investigated as an outcome in 15 studies (88.2%), wheezing in one study (5.9%), and wheezing with cold in another study (5.9%). The variables investigated in the selected studies as possible factors associated with wheezing were classified into the following dimensions, with their respective frequencies of investigation: **(1) Sociodemographic** – age (10/17, 58.8%), sex (9/17, 52.9%), family income (5/17, 29.4%), area of residence (5/17, 29.4%), region of the country (4/17, 23.5%), education (4/17, 23.5%), ethnic group (3/17, 17.6%), access to health services (3/17, 17.6%), home ownership (2/17, 11.7%), caregiver marital status (1/17, 5.9%), and caregiver occupation (1/17, 5.9%); **(2) Environmental** – smoking in the home (8/17, 47.0%), crowding (3/17, 17.6%), dampness or mold in the home (3/17, 17.6%), wood-burning stove in the home (3/17, 17.6%), pets in the home (2/17, 11.7%), insect and rodent infestation (2/17, 11.7%), endotoxin in the home (1/17, 5.9%) and **(3) Clinical-epidemiological** – asthma (15/17, 88.2%), allergy (5/17, 29.4%), RSV, protozoan, and bacterial ear infections (5/17, 29.4%), body mass index (4/17, 23.5%), breastfeeding (4/17, 23.5%), low birth weight (3/17, 17.6%), family history of asthma (3/17, 17.6%), atopy (2/17, 11.7%), prematurity (2/17, 11.7%), physical activity or exercise (2/17, 11.7%), and smoking during pregnancy (1/17, 5.9%).

The characteristics of the included studies and a summary of the data from this systematic review are presented in Table 1 (main text) and S1 Table (S6 Supplement).

**Table 1:** Characteristics of studies included in the systematic literature review.

First author/year	Location	Population				Study design	Outcome	Risk and bias classification
First author and publication year	Country	Ethnic group	Age (years)	Sample	N			
Kovesi 2022 [33]	Canada	FN	≤ 3	Community (Sioux Lookout)	98	Sectional	Wheezing with a cold	Low risk
Rennie 2020 [34]	Canada	FN	6-17	Community (Saskatchewan rural zone)	351	Sectional	Atopic and Non-Atopic Asthma	Low risk
Karunanayake 2020 [35]	Canada	A, M, I	12-19	Census	25,170	Sectional	Asthma	Low risk
Kinghorn 2019 [36]	USA	AI	6-17	Indigenous health service	323	Case-control	Asthma	Low risk
Best 2017 [37]	USA	AI	6-17	Indigenous health service	323	Case-control	Asthma	Low risk
Best 2016 [38]	USA	AI	6-17	Indigenous health service	324	Case-control	Asthma	Low risk
Senthilselvan 2016 [39]	Canada	FN	0-11	First Nations Regional Health Survey	6,657	Sectional	Asthma	Low risk
Overeem 2014 [40]	Venezuela	W	0-2	Warao community	229	Sectional	Recurrent wheezing	Low risk
Kraai 2013 [41]	Venezuela	W	2-10	Warao community	630	Sectional	Asthma	Low risk
Chang 2012 [42]	Canada	NAI, M, I, MA	6-14	Aboriginal People Survey (APS)	48,921	Sectional	Asthma	Low risk
Ye 2012 [43]	Canada	NAI, M, I, MA	0-6	Census	14,170	Sectional	Asthma	Low risk
Shepherd 2012 [44]	Australia	AI	0-17	Western Australian Aboriginal Child Health Survey	5,289	Sectional	Asthma	Low risk
Crighton 2010 [45]	Canada	A	0-14	Aboriginal People Survey	35,495	Sectional	Asthma	Low risk
Surdu 2006 [46]	USA	MN	2-14	St. Regis Mohawk Health Service Records	50	Case-control	Asthma	Low risk
Lewis 2004 [47]	USA	AN, AI	10-18	Community (Yukon-Kuskokwim)	377	Sectional	Asthma	Low risk
Schei 2004 [48]	Guatemala	Mayan	4-6	Rural communities (Western Highlands of Guatemala)	1,058	Sectional	Asthma	Low risk
Clarck 1995 [49]	USA	NA	3-13	Community (Jemez Pueblo)	318	Sectional	Asthma	Low risk

FN = First Nation; A = Aboriginal; M = Meties; I = Inuit; AI = American Indian; W = Warao; NAI = North American Indian; MA = Multiple Ancestries; AI = Australian Indigenous; MN = Mohawk; NA = Alaska Native; Mayan = Mam-speaking Native American population; NA = Native American.

Outcomes definitions varied substantially across studies. Most patients were diagnosed with physician-diagnosed asthma (n = 8, 47%), followed by the International Study of Asthma and Allergies in Childhood (ISAAC) questionnaire criteria (n = 5, 29%), International Classification of Diseases, Ninth Revision (ICD-9) diagnostic codes (n = 3, 18%), and symptom-based definitions (n = 1, 6%).

All selected articles used questionnaires to identify wheezing cases. Asthma, a major cause of wheezing in school-age children and adolescents, was defined as a parent-reported physician diagnosis of asthma” or parental perception of wheezing. Studies employing the ISAAC methodology have used standardized core questions about wheezing, physician diagnosis, other questionnaires, and physiological measures [50]. Obstructed airflow has long been recognized as the primary mechanism underlying wheezing, a key symptom of asthma and other diseases [51]. Wheezing can be considered an indicator of airway obstruction of interest, particularly for assessing young children and in situations where objective documentation of lung function is not readily available, such as among Indigenous populations [51]. However, this heterogeneity in outcome definitions limits the direct comparability of findings across studies.

### Summary of results

#### Assessment of study quality and risk of bias.

The seventeen studies (100%) showed high quality and low risk (≥8 points) of methodological bias. Analysis of the quality and risk of bias is available in a supplementary material (S2 Supplement).

### Factors investigated for association with wheezing

**(1) Sociodemographic factors**

Age was found to be a factor associated with wheezing in ten studies (58.8%); a statistically significant association with the outcome was identified in six (35.2%) of them [33,35,37,41,43,45]. There were variations in the age strata of the analyses and outcomes between the studies. Only one study [33] considered the outcome of wheezing associated with cold and identified an association between age < 3 years and wheezing in First Nations children living in an isolated community in Sioux Lookout, northwestern Canada. The remaining studies reported asthma as an outcome. The studies [35,43,45,49] identified a positive association between asthma and age in Indigenous children in North America, specifically in Canada. In contrast, another study [41] found an inverse association between age and asthma in the age ranges of 4–5 and 8–10 years. Therefore, wheezing combined with asthma was directly associated with age, whereas wheezing associated with cold was inversely associated with age.

Child’s sex was investigated in nine studies (52.9%), all of which identified a statistically significant association with the outcome. In five studies (29.4%), female sex was considered the exposure category [35,41,39,40,52]; in four of these (23.5%), a lower probability of wheezing was identified in girls than in boys. The remaining four studies [43,45,49,42] considered male sex as the exposure category; all of them found a positive association, indicating that the eight studies were consistent in identifying male patients as more likely to exhibit the outcome compared with female individuals.

Annual family income was assessed in five studies (29.4%); only two (11.8%) identified a positive association with the outcome [35,39]. However, income ranges in these studies were conflicting. One study [35] found a positive association between asthma and income below $44,999, while another study [39] found a positive association between asthma and high income of $60,000 or more.

The area of residence was investigated in five studies (29.4%). A positive association was identified between living in an urban area and asthma [35,45] in two studies (11.8%), while an inverse association was found between living in a rural area and asthma (42, 43) in two other studies (11.8%). Furthermore, although one study [39] did not show a statistically significant association between living in a non-isolated area and outcomes, the results suggest a higher risk in residents in these areas compared with those in isolated areas.

Four studies (23.5%) investigated the country’s regions; a positive association was identified, considering all regions investigated, in only one (5.9%), including the Atlantic/Quebec, Ontario, Manitoba/Saskatchewan, Alberta, and British Columbia [35]. Furthermore, an inverse association was identified between the Northern Territories and asthma in two studies (11.8%) [45,42].

Caregivers’ educational level was investigated in four studies (23.5%); contrary to expectations, a positive association was identified between higher educational level and the outcome of asthma [35] in only one study (5.9%). Ethnic group was investigated in three studies (17.6%); in only two (11.8%) of them, an inverse association was identified between asthma and Inuit ethnicity compared with other ethnicities studied, such as Metis, Multiple Ancestors, and North American Indians [43,42].

Access to health services was assessed in three studies (17.6%); only two (11.8%) of them identified an association with the outcome [43,42]. However, the study [42] showed a positive association between regular appointments and an increased prevalence of asthma, suggesting that children with more complex health conditions tend to seek medical care more regularly. In a previous study [43], the association between easy access to health services and asthma was inverse, suggesting that easy access to healthcare is associated with a lower probability of developing asthma. These studies complement our understanding about how various aspects of healthcare could influence the prevalence of asthma.

Two studies (11.8%) assessed home ownership; however, only one (5.9%) identified a positive association between financed and rented housing and asthma outcome of asthma [44]. Only one study assessed caregiver occupation and marital status (5.9%) and found no statistically significant association with asthma outcome [44,36].

**(2) Environmental factors**

Eight studies (47.0%) investigated smoking; a positive association between cigarette smoke exposure and the outcome of asthma was identified in three studies (17.6%) of them [35,41,52]. However, one study (5.9%) showed a positive association between the absence of smokers in the household and outcome of asthma, possibly because of uncontrolled confounding factors [39].

Three studies (23.5%) investigated household crowding; however, only two (11.8%) revealed a positive association between having 1–2 people under 18 and the outcome [35,39,53]. Furthermore, in the same study [39], a negative association was identified for people under 11 years of age in households with more than 4.

Three studies (23.5%) investigated moisture and mold formation. However, only one study (5.9%) identified a positive association between the lack of exposure and outcome of atopic asthma [34]. This observation contradicted expectations and suggested that it might have been influenced by other confounding factors that were not controlled in this study.

Three studies (17.6%) evaluated the use of wood-burning stoves at home; only one (5.9%) revealed a positive association between using wood-burning stoves as a cooking method and outcomes [41]. Two studies (11.8%) investigated the presence of a domestic animal; however, no statistically significant association was found between the presence of a domestic animal and outcome of asthma [36,46].

Two studies (11.8%) investigated insect, cockroach, and ant infestations, however, none showed a statistically significant association with asthma [36,46]. Only one study (5.9%) analyzed home exposure to endotoxins and identified a positive association with wheezing [33].

**(3) Clinical-epidemiological**

Allergy was investigated in five studies (29.4%); all identified a positive association with the outcome of asthma [37,42,36,53,38]. However, the presence of allergies was heterogeneously evaluated in these studies. Allergen sensitization, as identified by specific immunoglobulin E (IgE), was evaluated in two studies that referred to the same population [37,38]. Two studies inquired about the presence of allergies without precisely defining the term [42,53]. One study investigated atopy and food sensitization [36]. The term “potential atopy carrier” was used for describing individuals with rhinitis symptoms without cold, accompanied by conjunctival symptoms [47]. One study assessed allergen sensitization by performing a spot-read test for six allergens. However, this factor was not used as a risk factor but rather to stratify patients into two groups: atopic and non-atopic asthma [34]. Only one study evaluated genetic factors associated with asthma development and highlighted the importance of genetic variants in chromosomes 5q22.1 and 17q21 in Native American children [37]. Humoral factors, such as total serum IgE, peripheral eosinophils, and leukocytes were evaluated in only one study [38].

Five (29.4%) studies investigated previous infections. Three studies revealed a positive association between RSV, protozoan, and bacterial ear infections and asthma outcomes [43,40,36]. However, one study [53] showed no statistically significant association between chronic ear infection and asthma outcomes. Another study [34] showed that the prevalence of a history of respiratory infection was higher in the non-atopic asthma group.

Five studies (29.4%) investigated body mass index; four (23.5%) identified a positive association between being overweight or obese and asthma outcome [35,37,42,34]. However, one study (5.9%) did not find a statistically significant association with the outcome.

Four studies (23.5%) investigated breastfeeding; two (11.8%) showed an inverse association with breastfeeding [43,46]. Two other studies revealed no statistically significant associations with outcomes [49,42]. Three studies (17.6%) investigated low birth weight, all of which showed a positive association with outcomes [43,42,53].

Three studies (17.6%) investigated family history of asthma; two (11.8%) reported a positive association with asthma outcome [33.45]. Two studies (11.8%) investigated atopy; both revealed a positive association with asthma outcome [52,36].

Premature birth was investigated in two studies (11.8%); however, neither showed a statistically significant association with asthma outcome [33,46]. Physical activity and exercise were investigated in two studies (11.8%). One study (5.9%) of these two identified a positive association with asthma outcome [48], whereas another [53] showed an inverse association when exercising daily. Smoking during pregnancy was investigated in one study (5.9%); however, no statistically significant association with asthma outcome was identified [46].

## Discussion

This systematic review is the first to identify factors associated with wheezing in Indigenous children and adolescents by analyzing 17 observational studies with 139,783 participants from diverse ethnic groups across four continents.

### Clinical manifestations and diagnosis

Among the main findings in this study, asthma was identified as the most common condition for wheezing episodes (88.2%). A study comparing among Indigenous and non-Indigenous children in the prevalence of asthma included children aged 0–17 years, mostly <10 years, in five remote Indigenous communities in Australia and concluded that the prevalence of asthma and wheezing episodes was as high as in non-Indigenous groups [19]. Asthma remains one of the most common chronic respiratory diseases in children and is characterized by wheezing, shortness of breath, and coughing, which can be triggered by various stimuli. Wheezing is a key feature for diagnosing asthma. If it is not present, it is unlikely that a diagnosis of asthma will be made in a child [54]. However, several other causes of wheezing in childhood, including cystic fibrosis, gastroesophageal reflux disease, bronchiolitis, and parasitosis appear to be underdiagnosed [55].

Male sex was associated with a higher incidence of wheezing as evidenced in eight articles, whereas one study showed a risk associated with female sex. An asthma prevalence study that included 1,290 urban Aboriginal children aged 1–17 years and found a higher prevalence of asthma among boys [56]. However, in the scientific literature, a disparity between the sexes regarding asthma is apparent, with a higher prevalence in boys up to 13 years of age (65%). This pattern changes in adulthood, when asthma becomes more prevalent in female individuals. This male predominance aligns with the global trends in pediatric asthma [57]. The only study that identified a risk associated with female sex studied a population aged 10–18 years, of which 33% were over 14 years of age, which might have influenced this result [47].

### Socioeconomic factors

Regarding annual income, one study found an association between high income and the risk of wheezing [39], whereas two others found an association with low income [58,59]. The association between wheezing and higher income was attributed to the fact that these parents also had higher levels of education and better access to healthcare and diagnostic services [39]. Conversely, a study that found a higher occurrence of wheezing among those with lower incomes reported greater vulnerability to viral infections, which were triggers of asthma in this population, as well as a higher rate of hospitalization due to asthma [59].

Living in rural areas was considered a protective factor. The lower incidence of wheezing among rural residents was attributed to the hygiene hypothesis, originally proposed by Strachan in 1989 [60], which posits that reduced microbial exposure in modern environments increases susceptibility to allergic diseases. Subsequent studies in general populations have consistently documented that agricultural environments, characterized by high exposure to microbial agents such as endotoxins and diverse bacterial communities, confer a protective effect against asthma and atopy [61,62,63]. Similar protective effects of rural environments have been observed in indigenous populations [58]. Some authors have reported a higher prevalence of asthma symptoms in children living in urban areas, which might be attributed to the protection of the agricultural environment [61,64,65]. Greater exposure to pollutants in urban environments might also contribute to this protective rural effect, which usually produces fewer pollutants than urban environments [45]. However, one factor that might contribute to a non-beneficial protective effect is reduced access to health services in rural areas, and consequently, lower identification of wheezing cases in these areas [66].

### Environmental factors

Investigating specific rural exposures such as microbial diversity might provide insights into reducing asthma rates in urban environments. Identifying and replicating these protective factors could inform policies and support targeted public health interventions, ultimately enhancing their effectiveness.

Tobacco exposure revealed contradictory results, with studies associating a greater magnitude of wheezing among individuals who had no contact with passive smokers. As the harmful effects of tobacco exposure have already been proven in scientific literature to exacerbate asthma and other respiratory diseases [67], the higher likelihood of wheezing in an environment without tobacco exposure could be interpreted as a consequence and not a cause, reflecting the behavior of adults who stop smoking because they have an asthmatic child at home. Regarding passive smoking exposure, we observed that two studies reported it differently, which hindered our comparison, highlighting, not only in this variable but also in several others, the importance of standardizing some exposure indicators besides wheezing-related outcomes to identify modifiable risk factors and change the pattern of occurrence of wheezing, asthma, and their repercussions on children’s health.

Two studies have identified exposure to wood-burning stoves as a risk factor for wheezing. An integrative review of the literature to determine the environmental factors contributing to the increased prevalence and severity of asthma among Navajo children living on reservations identified indoor air pollution, with particular emphasis on exposure to wood-burning stoves and coal combustion, as factors that increase the risk of respiratory disease [68].

Biomass fuels used for heating homes and cooking, as well as cigarette and wood-burning stove smoke, contain numerous human carcinogens, including polycyclic aromatic hydrocarbons, which have higher levels of carbonaceous nuclei than cigarette smoke [69]. To provide clearer intervention targets, specifying particulate matter (PM2.5) levels or polycyclic aromatic hydrocarbon concentrations in wood-burning stoves could offer readers an actionable framework for environmental regulations. These exposures might be associated with several acute and chronic respiratory diseases, such as asthma, acute respiratory tract infections in adults and children, chronic obstructive pulmonary disease, lung cancer, and tuberculosis [70].

Studies have suggested that caregivers believe that protecting children from exposure to secondhand tobacco smoke can be an important motivation for establishing smoke-free homes [71]. Thus, pollution within the home environment should be assessed, along with the beliefs of the Indigenous population, which can help identify barriers to achieving a smoke-free environment.

Among other housing conditions, such as the presence of mold, humidity, and endotoxins, only exposure to endotoxins was significantly associated with the outcome. The authors suggested that the increased risk of wheezing associated with cold, when combined with exposure to endotoxin, a component of the cell wall of Gram-negative bacteria, might indicate that asthma has been underdiagnosed [72]. The use of a self-report questionnaire in a study assessing the presence of mold and humidity might have underestimated the actual presence of these factors, which could have affected the final results. This highlights the importance of a more objective assessment [73] of these potential exposure risks. A systematic review of the European Academy of Allergy and Clinical Immunology guidelines on environmental science for allergic diseases and asthma identified that exposure to moisture or mold increases the risk of wheezing [74]. Standardizing the collection of these data using the Environmental Relative Moldiness Index might provide more accurate information on this risk factor [75].

Access to health services is important for interpreting data on the risk factors for wheezing. However, only one study has evaluated this issue; univariate analysis showed that the prevalence of asthma among children with difficulty in accessing health services was twice as high as that among those without the same barriers to access [76].

### Early-life and nutritional factors

Low birth weight was assessed in three studies and found to be positively associated with asthma, whereas breastfeeding during infancy was identified as a protective factor. Together, these data indicate the importance of perinatal care in preventing asthma development in the first months of life. An association between being overweight and/or obese and asthma was found in four studies and has already been indicated in the literature as having a bidirectional association [77]. Obesity might result in a decreased response to inhaled corticosteroids owing to changes in airway smooth muscle and restricted chest wall movement, in addition to chronic low-grade systemic inflammation, leading to more frequent asthma symptoms. Conversely, poorly controlled asthma might result in limited physical activity and lead to a sedentary lifestyle, which contributes to obesity [77].

### Allergic and immunological factors

Regarding allergies, lacking standardization in defining and measuring exposure has hindered the comparison of studies. Allergen sensitization to aeroallergens, food allergens, and rhinitis in the absence of cold are among the criteria that contribute to the score for predicting modified asthma development in preschool children [78]. Scores for predicting asthma in certain age groups that might be useful for this assessment are already available [79]. Identifying other allergic diseases associated with wheezing, such as rhinitis, atopic dermatitis, allergic conjunctivitis, and food allergies, might increase the probability of allergic asthma due to atopic diathesis [80]. To improve comparability among future studies, researchers should consider using standardized tools and criteria such as validated questionnaires and biomarker panels for allergy assessment. Such tools could help ensure consistent data collection and a more robust analysis of allergic risk factors.

### Genetic factors

Regarding the genetic risks for developing asthma, only one study contributed to increasing the knowledge about these factors in the indigenous population, which has historically been excluded from genetic studies of asthma [37].

### Infectious factors

The predisposition to wheezing associated with respiratory infections should be understood in a broader context. Therefore, an important concept is that of a single airway, in which the upper and lower airway management must be understood as a single physiological unit, meaning that an inflammatory condition affecting one system tends to affect another similarly [81]. Because of the connection of the middle ear to the airway via the Eustachian tube, middle otitis might increase the risk of asthma, particularly in children, due to the immaturity of the auditory or Eustachian tube [82].

Another important factor to consider is that, although asthma is a significant cause of wheezing in childhood, many of our children experience wheezing as infants, typically during viral infections. Cohort studies conducted in the US [83,84], Australia [85,86], and Europe [87] have shown that although wheezing is common during childhood, specifically in association with respiratory infections, it usually resolves spontaneously by the age of 3 years. Determining whether wheezing during a viral infection is an early manifestation of asthma is challenging [88]. Understanding whether wheezing caused by viral infections, which are most common in childhood, is underdiagnosed and interpreted as asthma is crucial. Severe RSV infection early in life is associated with wheezing onset in childhood [89]. Studies by Sigurs et al. (2010) showed a 7.2-fold increased risk of asthma at the age of 18 years following severe RSV bronchiolitis [90]. Epidemiological studies have suggested that the risk of asthma following bronchiolitis is related to the episode’s severity, with children requiring hospitalization showing a 2.8-fold increased risk of asthma at ages 4.5–5.5 [91]. Bacharier et al. (2012) also demonstrated that severe RSV bronchiolitis in the first year of life is associated with a diagnosis of asthma around the age of 7 years in 48% of children, corresponding to nearly half of all children [92]. Vaccination of pregnant women at the end of pregnancy with RSV reduces hospitalizations in children [93]; moreover, its protective effect against asthma might be promising. However, the results of these studies were unclear.

### Parasitic infections

Overeen et al. (2014) [40] found a protective effect associated with parasitic infection, which has already been described in the literature and is attributed to the immunomodulatory effect of infections by parasites such as *A. lumbricoides*, *Schistosoma mansoni*, *Strongyloides stercoralis*, and *T. trichiura*, which have been associated with reduced allergic airway inflammation [94]. Despite this, Loeffler’s syndrome, a transient eosinophilic pneumonia caused by helminths, should be considered in the differential diagnosis of wheezing in childhood [95,96].

### Geographical disparities and research gaps

However, 76.4% of these studies were conducted in North America, a region comprising a small proportion of the global Indigenous population. This geographic concentration highlights the systemic research funding and priority patterns that limit the representation of broader ethnic and geographic diversity. Addressing this imbalance requires equitable research funding for underrepresented regions as well as collaboration with local researchers and Indigenous communities. Translating social drivers, such as ‘income inequality’ and ‘housing quality,’ into quantifiable metrics that allow these factors to be consistently embedded across studies is essential. This operational definition will guide future researchers in effectively integrating equity variables.

To address geographic research gaps, a concrete next step could be to initiate region-specific funding programs that encourage research participation from underrepresented areas such as Central and South America and Oceania. These programs can be designed to support local institutions and provide resources for the comprehensive study of Indigenous populations. In addition, organizing joint research initiatives and exchange programs between universities in well-researched regions and institutions in underrepresented areas can foster collaboration and mutual knowledge transfer.

Capacity building and knowledge transfer initiatives are essential for achieving more comprehensive and representative research. To facilitate this, specific strategies such as training workshops and research partnerships with local Indigenous communities and institutions are crucial. Establishing platforms for researcher exchange and providing funding for collaborative projects can also promote knowledge transfer and build local research capacity, paving the way for more inclusive and diverse studies worldwide. Additionally, implementing dedicated funding mechanisms such as grants specifically for indigenous health research can effectively prioritize this area. Developing partnership models that involve government agencies, universities, and community organizations can also contribute to a more coordinated approach to address these challenges. Ultimately, establishing an international consortium dedicated to indigenous respiratory health research might unify global efforts and ensure sustained attention and effective allocation of resources.

According to statistical data from the Brazilian Institute of Geography and Statistics for the 2022 Census, the Brazilian Indigenous population is estimated to be approximately 1.7 million (representing 305 ethnic groups), which accounts for 0.83% of the national population and is mainly concentrated in the northern and northeastern regions of the country. However, no Brazilian study has been identified that evaluates factors associated with wheezing in isolation, that is, without considering the context of other respiratory diseases and their severity, such as hospitalization and death, as outcomes [27,30,31,97,98]. To address these regional research gaps, it is crucial to implement targeted strategies, such as increased funding specifically aimed at respiratory health research, among Indigenous Brazilian communities. Establishing partnerships with local universities, health institutes, and indigenous organizations is essential for initiating and sustaining these research efforts. One practical approach is to organize co-design workshops with Indigenous Brazilian leaders to collaboratively develop future studies. These workshops could incorporate participatory methods, such as focus group discussions and scenario planning, to ensure that the research questions and methodologies align with the needs and insights of Indigenous communities. Initiatives such as research grants and scholarships for studies focused on respiratory health issues affecting Indigenous populations could further stimulate interest and expertise in this under-researched field. Encouraging collaboration and data sharing among regional and international researchers may also enhance the quality and scope of research findings, ultimately fostering a more comprehensive understanding of the determinants of wheezing in these communities.

### Study limitations

A significant limitation of this systematic review was the geographical distribution of the included studies, with 76.5% conducted in North America. This geographical concentration might compromise the generalizability of the results to other regions, particularly in low- and middle-income countries, where socioeconomic conditions, health systems, and population characteristics might present substantial disparities.

The predominance of North American studies could be attributed to various factors, including the greater availability of research resources, differences in scientific publication practices, and selection biases related to the databases consulted. The unequal distribution of this information compromises our ability to understand how these findings apply to contexts with different health infrastructures, cultural variations, distinct epidemiological profiles, and limited economic resources.

Furthermore, the studied populations may not be representative of global ethnic and racial diversity, which might have affected the external validity of the results. Disparities in treatment protocols, clinical guidelines, and health financing systems across countries might also have influenced the observed outcomes, thereby limiting the international applicability of the findings.

This geographical limitation constitutes a significant gap in current knowledge, which underscores the urgent need for additional studies conducted in underrepresented regions. In this study, we aimed to validate and expand our understanding about the factors associated with wheezing in Indigenous children and adolescents across various global contexts. In future systematic reviews, more comprehensive search strategies should be prioritized, and the inclusion of regional databases should be emphasized to reduce geographical bias.

Beyond geographical limitations, this systematic review encountered several methodological challenges, primarily related to study heterogeneity, which precluded conducting a meta-analysis.

First, there was substantial variability in the definitions of the outcomes across studies. The definitions ranged from standardized ISAAC questionnaires (23.5%) and physician diagnoses (41.2%) to administrative ICD-9 codes (17.6%) and symptom-based self-reports (11.8%). This definitional heterogeneity might have influenced case identification and the strength of the associations with risk factors.

Second, lacking standardization in defining and collecting data on potential risk factors, whether wheezing alone or in conjunction with asthma and bronchiolitis, represents a significant challenge for evidence synthesis. This variability limits the ability to identify modifiable factors that can be targeted through interventions to reduce the burden of wheezing and asthma in Indigenous children and adolescents.

Standardization of outcome definitions and risk factor measurements in future studies would substantially enhance comparability and enable robust meta-analyses in Indigenous populations.

Another limitation is the design of the identified studies, which was predominantly restricted to cross-sectional and case-control studies. Such studies prevent the calculation of direct risk averages and are more susceptible to biases, such as reverse causality and recall bias, which have the potential to distort the investigated relationships. This result is noteworthy, considering the satisfactory quality assessment of the articles included in the review. To address these limitations, prioritizing the implementation of prospective cohort studies is imperative, particularly in underrepresented regions, to establish a clearer temporal sequence and causality. Prospective birth cohort studies can monitor participants from birth and collect exhaustive data on salient variables such as smoke exposure, wheezing incidence, and other potential risk factors over time. Thus, a two-stage case-control study would be beneficial. In this study, cases and controls were matched based on specific criteria, and retrospective data on previous smoke exposure were collected. Furthermore, the incorporation of mixed-methods or participatory research designs has the potential for addressing existing limitations by combining quantitative data with qualitative insights, thereby providing a more comprehensive understanding of wheezing across diverse contexts.

To address the limitations of this review, we recommend several research priorities.

First, prospective cohort studies in underrepresented regions, particularly Central and South America, Oceania, Asia, and Africa, are urgently needed for establishing temporality and enabling causal inferences regarding environmental, socioeconomic, and genetic factors associated with wheezing. Priority research questions for such studies include: (1) whether early life tobacco smoke and indoor air pollution exposure increase the risk of developing wheezing by the age of five, as measured by pulmonary function tests; (2) how variations in socioeconomic status (family income, healthcare access, housing quality) predict differences in wheezing prevalence and severity across diverse indigenous populations; and (3) identification of specific genetic markers associated with elevated wheezing risk in indigenous populations and their potential utility for targeted prevention strategies.

Second, methodological standardization is essential. Future studies should adopt standardized outcome definitions (such as the ISAAC questionnaire criteria) and consistently measure exposure variables (including objective assessments of indoor air quality, mold exposure using the Environmental Relative Moldiness Index, and validated socioeconomic indicators). This standardization enables robust meta-analyses and more definitive conclusions about modifiable risk factors. Incorporating community consent processes into the study design is crucial to ensure that participatory ethics are an integral part of the research methodologies, thereby safeguarding cultural safety throughout the research process.

Third, community-based participatory research approaches are appropriate for indigenous populations. Longitudinal projects that involve Indigenous communities in the conception, design, and implementation of studies ensure cultural appropriateness and ethical conduct while addressing local priorities. Mixed-method studies, which combine quantitative epidemiological data with qualitative interviews, can capture cultural and contextual factors that influence respiratory health, which purely might be overlooked by quantitative.

Finally, specific attention should be directed toward underdiagnosed conditions, such as parasitic infections, severe RSV bronchiolitis, and non-asthmatic causes of wheezing in indigenous children. Understanding the full spectrum of wheezing etiologies in these populations is essential for developing comprehensive and culturally appropriate prevention and management strategies.

## Conclusion

In this systematic review we summarize evidence from 17 studies on factors associated with wheezing in Indigenous children and adolescents globally, identifying three main domains of determinants: environmental factors (tobacco smoke exposure, indoor air pollution from wood-burning stoves, and housing conditions, including mold and humidity), socioeconomic factors (income level, healthcare access, and rural-urban residence), and biological/clinical factors (male sex, age, low birth weight, obesity, breastfeeding, allergic sensitization, respiratory infections, and parasitic infections).

Based on these findings, we proposed a theoretical model to analyze the determinants of wheezing in Indigenous Brazilian children, accounting for the complex interplay among these three domains within the specific context of Brazil’s 305 indigenous ethnic groups.

Wheezing in Indigenous children is not solely a clinical issue, but a reflection of structural and environmental determinants. Our systematic review identified three key domains: environmental exposure (tobacco smoke, biomass fuel, and mold), socioeconomic inequities (low income and rural healthcare barriers), and biological susceptibility (early life respiratory infections and male sex). Although the data align with global asthma patterns, the specific burden of indoor air pollution and housing quality in Indigenous communities highlights urgent targets for public health intervention.

However, the generalizability of these findings is constrained by a significant research bias toward North American populations and the absence of data from the Global South, particularly Brazil, where the interplay between infectious and allergic wheezing remains poorly understood. To address this issue, future research must move beyond cross-sectional designs to prospective cohorts with standardized definitions of wheezing and environmental exposure.

Validating this model requires prospective cohort studies in Brazil that use standardized definitions and community-based participatory approaches to address local cultural and socioeconomic realities. The proposed theoretical model, integrating the environmental, socioeconomic, and biological domains, should guide future studies to identify unique respiratory health determinants for Indigenous children in underrepresented regions.

## Supporting information

S1 Supplement Database search. (DOC)

S2 Supplement Quality and risk of bias analysis. (DOC)

S3 Supplement PRISMA 2020 checklist. (PDF)

S4 Supplement PRISMA 2020 for abstracts checklist. (PDF)

S5 Supplement Prospero Record. (PDF)

S6 Supplement S1 Table: Summary of results. (DOCX)
