# Supplementary material for: Factors associated with wheezing in Indigenous children and adolescents: A systematic review of the global literature
Source: PLoS One. 2026 Mar 27;21(3):e0345711. doi: 10.1371/journal.pone.0345711 (PMC13029807; doi:10.1371/journal.pone.0345711)
Supplement: S1 Supplement — (DOC) [file pone.0345711.s001.doc]

**ARTICLE SEARCH**

**PUBMED**

((wheezing OR bronchiolitis OR bronchospasm OR asthma) AND ((“indigenous infants”) OR (“native children" ) OR ( "indigenous children" ) OR ( "indigenous population" ) OR ( "Indians central American" ) OR ( "Indians north american" ) OR ( "Indians south american" ) OR ( "health of indigenous peoples" ) OR ( "aborigines Australian" ) OR ( "first nation" ))) AND (pediatric OR infants OR children) AND (risk factors OR associate factors OR etiology OR causes OR protection factors).

**SCOPUS**

( TITLE-ABS-KEY ( wheezing OR bronchiolitis OR bronchospasm OR asthma ) AND TITLE-ABS-KEY ( ( "indigenous infants" ) OR ( "native children" ) OR ( "indigenous children" ) OR ( "indigenous population" ) OR ( "indians central american" ) OR ( "indians north american" ) OR ( "indians south american" ) OR ( "health of indigenous peoples" ) OR ( "aborigines australian" ) OR ( "first nation" ) ) AND TITLE-ABS-KEY ( pediatric OR infants OR children ) AND TITLE-ABS-KEY ( ( risk AND factors OR associate AND factors OR etiology OR causes OR protection AND factors ) ) ).

**LILACS**

((wheezing OR bronchiolitis OR brochospasm OR asthma) AND ((“indigenous infants") OR (“native children”) OR ( "indigenous children" ) OR ( "indigenous population" ) OR ( "indians central american" ) OR ( "indians north american" ) OR ( "indians south american" ) OR ( "health of indigenous peoples" ) OR ("aborigines australian" ) OR ( "first nation" ))) AND (pediatric OR infants OR children) AND (risk factors OR associate factors OR etiology OR causes OR protection factors).

**WEB OF SCIENCE**

(wheezing OR bronchiolitis OR bronchospasm OR asthma) AND (“indigenous infants" ) OR ( "native children" ) OR ( "indigenous children" ) OR ( "indigenous population" ) OR ( "Indians central American" ) OR ( "indians north american" ) OR ( "indians south American" ) OR ( "health of indigenous peoples" ) OR ( "aborigines australian" ) OR ( "Fisrt Nation" ) (Todos os campos) AND (pediatric OR infants OR children) AND (risk factors OR associate factors OR etiology OR causes OR protection factors).
