# Supplementary material for: Factors associated with wheezing in Indigenous children and adolescents: A systematic review of the global literature
Source: PLoS One. 2026 Mar 27;21(3):e0345711. doi: 10.1371/journal.pone.0345711 (PMC13029807; doi:10.1371/journal.pone.0345711)
Supplement: S2 Supplement — (DOC) [file pone.0345711.s002.doc]

**QUALITY ASSESSMENT AND RISK OF BIAS OF THE STUDIES**

**Table 1. Quality assessment of the case-control studies included in the systematic review, using the Newcastle-Ottawa Scale.**

| **First author, year of publication and country**  **Selection** | **Selection**  **(maximum 4)** | | | | **Comparability**  **(maximum 2)** | **Exposure/result**  **(maximum 3)** | | | **Total**  **(maximum 9)** |
| --- | --- | --- | --- | --- | --- | --- | --- | --- | --- |
| Kinghorn, 2019, EUA | * | * | * | * | ** | * | * | * | 9 |
| Best, 2017, EUA | * | * | * | * | ** | * | * | * | 9 |
| Best, 2016, EUA | * | * | * | * | ** | * | * | * | 9 |
| Surdu, 2006, EUA | * | * | * | * | * | * | * | * | 8 |

**1-Selection:** a) Adequacy of case definition. b) Representativeness of cases. c) Selection of controls. d) Definition of controls. **2-Comparability:** a) Comparability of cases and controls based on study design or analysis. **3- Exposure/Outcome:** a) Determination of exposure. b) Same method of determination for cases and controls. c) Non-response rate.

**Table 2 - Quality assessment of the cross-sectional studies included in the systematic review, using the Newcastle-Ottawa Scale.**

| **First author, year of publication and country**  **Selection** | **Selection**  **(maximum 5)** | | | | | **Comparability**  **(maximum 2**) | **Exposure/result**  **(maximum 2)** | | | **Total**  **(maximum 9)** |
| --- | --- | --- | --- | --- | --- | --- | --- | --- | --- | --- |
| Kovesi, 2022, Canadá | * | * | * | * | * | * | * | * |  | 8 |
| Rennie, 2020, Canadá | * | * | * | * | - | ** | * | * |  | 8 |
| Karunanayake, 2020, Canadá | * | * | * | * | * | * | * | * |  | 8 |
| Senthilselvan, 2015, Canadá | * | * | * | * | * | * | * | * |  | 8 |
| Overeem, 2014, Venezuela | * | * | * | * | * | * | * | * |  | 8 |
| Kraai, 2013, Venezuela | * | * | * | * | * | * | * | * |  | 8 |
| Chang, 2012, Canadá | * | * | * | * | * | ** | * | * |  | 9 |
| Shepherd, 2012, Austrália | * | * | * | * | * | * | * | * |  | 8 |
| Ye, 2012, Canadá | * | * | * | * | * | ** | * | * |  | 9 |
| Crighton 2010, Canadá | * | * | * | * | * | ** | * | * |  | 9 |
| Lewis, 2004, EUA | * | * | * | * | * | * | * | * |  | 8 |
| Schei, 2004, Guatemala | * | * | * | * | * | * | * | * |  | 8 |
| Clark, 1995, EUA | * | * | * | * | * | * | * | * |  | 8 |

**1-Selection:** a) Sample representativeness. b) Sample size. c) Sample selection. d) Definition of exposure and outcome. e) Non-response rate. **2-Comparability:** a) Confounding factor control. **3-Exposure/Outcome:** a) Exposure/outcome assessment. b) Same methodology for all participants.
