# Supplementary material for: Factors associated with wheezing in Indigenous children and adolescents: A systematic review of the global literature
Source: PLoS One. 2026 Mar 27;21(3):e0345711. doi: 10.1371/journal.pone.0345711 (PMC13029807; doi:10.1371/journal.pone.0345711)
Supplement: S5 Supplement — (PDF) [file pone.0345711.s005.pdf]

PROSPERO
International prospective register of systematic reviews

A systematic review of the world literature on factors associated with wheezing in Indigenous children and adolescents

Marcia Corrêa de Castro, Daniella Moore, Andrey Cardoso, Saint Clair Junior, Karla Camacho, Zina Maria Almeida de Azevedo, Sandra Lisboa

To enable PROSPERO to focus on COVID-19 submissions, this registration record has undergone basic automated checks for eligibility and is published exactly as submitted. PROSPERO has never provided peer review, and usual checking by the PROSPERO team does not endorse content. Therefore, automatically published records should be treated as any other PROSPERO registration. Further detail is provided **here**.

Citation

Marcia Corrêa de Castro, Daniella Moore, Andrey Cardoso, Saint Clair Junior, Karla Camacho, Zina Maria Almeida de Azevedo, Sandra Lisboa. A systematic review of the world literature on factors associated with wheezing in Indigenous children and adolescents. PROSPERO 2023 CRD42023395661 Available from: https://www.crd.york.ac.uk/prospero/display_record.php?ID=CRD42023395661

Review question

What factors are associated with wheezing in indigenous children and adolescents?

P - Indigenous children and adolescents
I/E - Factors are associated with wheezing
C - Indigenous children and adolescents without wheezing
O - Wheezing
S - Prevalence, Cross-sectional, Cohort, and Case-Control Studies

Searches

PubMed; Web of Science Scopus and LILACS. Search date March 6, 2023. No restriction on language or publication date.

Types of study to be included

Observational studies such as cohort, prevalence, cross-sectional study, and case-control studies. Exclusion of review studies and meta-analysis.

Condition or domain being studied

Wheezing. Bronchiolitis. Asthma. Indigenous Children and Adolescents.

Participants/population

Inclusion: studies in indigenous children and adolescents that evaluate risk factors for wheezing; without restriction of publication languages.

Exclusion: studies that did not present a description of wheezing or did not present separate results for indigenous people; editorials, letters, review studies, and conference proceedings; studies in which

wheezing was related to chronic lung disease and bronchiectasis; articles not available in full; articles that did not present a design that would allow the assessment of risk factors.

Intervention(s), exposure(s)

In the world literature, there are several exposures (risk and protection factors) for wheezing. However, for indigenous crildren, these exposures are unknown.

Comparator(s)/control

Indigenous children and adolescents without wheezing.

Context

Studies on so-called indigenous or native populations anywhere in the world.
No limits were included for research, avoiding excessive specifications such as type of studies or year of publication, in order to maximize the review of articles, due to the scarcity of studies on indigenous populations, mainly in Brazil.

Main outcome(s)

Any protection or risk factors that may be associated with wheezing in indigenous children and adolescents of the world.

Additional outcome(s)

None

Data extraction (selection and coding)

The studies found were imported into the Covidence program and the identification and exclusion of duplicates was performed. Screening of titles and abstracts, as well as a selection of full text, data extraction, and quality analysis, will be carried out by two independent researchers and, when necessary, agreed upon by a third researcher.

Risk of bias (quality) assessment

Two review authors will independently assess the risk of bias in included studies by considering the following characteristics: Purpose of the study, Conflict of interests, Description of the population, Participant recruitment method, Control for confounding factors, and Appropriate statistical analysis.

Disagreements between the review authors over the risk of bias in particular studies will be resolved by discussion, with the involvement of a third review author where necessary.

Strategy for data synthesis

We will provide a descriptive summary of the results of the included studies, structured around the category of the associated factors, such as prenatal, socioeconomic, environmental, and biological factors, among others.
We anticipate that there will be limited scope for meta-analysis because of the range of different factors measured through the small number of existing studies. However, where studies have evaluated the same type of factors associated with the same outcome, we will assemble the results using a random effects meta-analysis, with standardized mean differences for continuous outcomes and risk ratios for binary outcomes, and calculate 95% Confidence Intervals.
Let us consider a two-value value greater than 50% indicative of substantial heterogeneity. We will perform sensitivity analysis based on study quality. We will use stratified meta-analyses to explore the heterogeneity

in effect estimates according to the quality of the study. We will also evaluate the evidence of publication bias.

Analysis of subgroups or subsets

While subgroup analysis may be undertaken it is not possible to specify the groups in advance.

Contact details for further information

Marcia Castro
marcia-castro-mc@fiocruz.br

Organisational affiliation of the review

National Institute of Women's, Children's and Adolescents' Health Fernandes Figueira - Oswaldo Cruz Foundation
https://portal.fiocruz.br/

Review team members and their organisational affiliations

Ms Marcia Corrêa de Castro. Fundação Oswaldo Cruz
Dr Daniella Moore. Fundação Oswaldo Cruz
Dr Andrey Cardoso. Fundação Oswaldo Cruz
Dr Saint Clair Junior. Fundação Oswaldo Cruz
Dr Karla Camacho. Fundação Oswaldo Cruz
Dr Zina Maria Almeida de Azevedo. Fundação Oswaldo Cruz
Dr Sandra Lisboa. Fundação Oswaldo Cruz

Type and method of review

Systematic review

Anticipated or actual start date

06 March 2023

Anticipated completion date

30 November 2023

Funding sources/sponsors

National Institute of Women's, Children's and Adolescents' Health Fernandes Figueira - Oswaldo Cruz Foundation

Conflicts of interest

Language

(there is not an English language summary)

Country

Brazil

Stage of review

Review Ongoing

Subject index terms status

Subject indexing assigned by CRD

Subject index terms

Adolescent; Asthma; Case-Control Studies; Child; Cross-Sectional Studies; Humans; Prevalence; Respiratory Sounds

Date of registration in PROSPERO

07 July 2023

Date of first submission

26 June 2023

Stage of review at time of this submission

Stage	Started	Completed
Preliminary searches	Yes	No
Piloting of the study selection process	Yes	Yes
Formal screening of search results against eligibility criteria	Yes	No
Data extraction	No	No
Risk of bias (quality) assessment	No	No
Data analysis	No	No

The record owner confirms that the information they have supplied for this submission is accurate and complete and they understand that deliberate provision of inaccurate information or omission of data may be construed as scientific misconduct.

The record owner confirms that they will update the status of the review when it is completed and will add publication details in due course.

Versions

07 July 2023
07 July 2023

PROSPERO
This information has been provided by the named contact for this review. CRD has accepted this information in good faith and registered the review in PROSPERO. The registrant confirms that the information supplied for this submission is accurate and complete. CRD bears no responsibility or liability for the content of this registration record, any associated files or external websites.
