# Supplementary material for: Factors associated with wheezing in Indigenous children and adolescents: A systematic review of the global literature
Source: PLoS One. 2026 Mar 27;21(3):e0345711. doi: 10.1371/journal.pone.0345711 (PMC13029807; doi:10.1371/journal.pone.0345711)
Supplement: S6 Supplement — (DOCX) [file pone.0345711.s006.docx]

**S1 Table. Summary of study results and associated effect measures**

| **Author** | **Outcome (Definition)** | **Exposure** | **Association measure** | **Confidence Interval (CI%)** | |
| --- | --- | --- | --- | --- | --- |
| **Kovesi, 2022** | Wheezing with a cold in the age group ≤ 3  of FN  (questionnaire-based symptom report) | Endotoxin unit per square meter (log endotoxin, EU/m^2^) | Odds Ratio | **1.32 (95% 1.04–1.70)** | |
|  |  | Age (years) |  | **1.70 (95% 1.03–2.90)** | |
|  |  | Preterm birth |  | Yes: 1.09 (95% 0.32–3.62) | |
|  |  | Particulate Matter <2.5µg/m^3^ |  | 0.99 (95% 0.98–1.00) | |
|  |  | Mold surface area > 0.2m^2^ |  | Yes: 0.82 (95% 0.20–3.02) | |
|  |  | Carbon Dioxide ppm (measured in indoor air) |  | 1.00 (95% 0.99–1.00) | |
| **Rennie, 2020** | Atopic and Non–Atopic Asthma  in the age group of 6–17 years of FN (physician diagnosis or wheeze in past 12 months; atopy assessed by SPT) | **Moisture harm** | | |  |
|  |  | BMI | Odds Ratio | Atopic Asthma  Obese: **5.9 (95%** **1.31–26.34**);  Overweight: 1.5 (95% 0.32–6.96)  Non–Atopic Asthma  Obese: 1.6 (95% 0.67–3.65);  Overweight: 1.1 (95% 0.51–2.22) |  |
|  |  | Had infection sometimes |  | Atopic Asthma  No: 0.6 (95% 0.17–2.02)  Non–Atopic Asthma  No: **3.6 (95%** **1.65–7.65)** |  |
|  |  | Passive smoker |  | Atopic Asthma  No: 1.7 (95% 0.38–5.69)  Non–Atopic Asthma  No: 1.8 (95% 0.90–3.68) |  |
|  |  | Fuel type |  | Atopic Asthma  Natural gas: 2.8 (95% 0.55–14.38)  Non–Atopic Asthma  Natural gas: **4.5 (95%** **1.44–14.06)** |  |
|  |  | Moisture harm |  | Atopic Asthma  No: **5.5 (95%** **1.43–20.99)**  Non–Atopic Asthma  No: 1.2 (95% 0.60–2.38) |  |
|  |  | **Presence of fungus or mold** | | |  |
|  |  | BMI | Odds Ratio | Atopic Asthma  Obese: **6.8 (95%** **1.54–29.91)**; Overweight: 1.6 (95% 0.36–7.42)  Non–Atopic Asthma  Obese:1.6 (95% 0.69–3.75); Overweight: 1.1 (95% 0.54–2.32) |  |
|  |  | Had infection sometimes |  | Atopic Asthma  No: 0.7 (95% 0.19–2.15)  Non–Atopic Asthma  No: **3.7 (95%** **1.73–8.03)** |  |
|  |  | Passive smoker |  | Atopic Asthma  No: 1.7 (95% 0.47–6.38)  Non–Atopic Asthma  No: 2.0 (95% 0.98–3.89) |  |
|  |  | Fuel type |  | Atopic Asthma  Natural gas: 2.6 (95% 0.50–13.53)  Non–Atopic Asthma  Natural gas: **4.3 (95%** **1.37–13.26)** |  |
|  |  | Fungus or mold signs |  | Atopic Asthma  No: 3.6 (95% 0.99–12.91)  Non–Atopic Asthma  No: 0.8 (95% 0.41–1.55) |  |
|  |  | **Moisture in the house in the last 12 months** | | |  |
|  |  | BMI | Odds Ratio | Atopic Asthma  Obese: **8.9 (95%** **2.00–39.11)**; Overweight: 1.9 (95% 0.41–8.46)  Non–Atopic Asthma  Obese: 1.6 (95% 0.70–3.71); Overweight: 1.1 (95% 0.51–2.22) |  |
|  |  | Had infection sometimes |  | Atopic Asthma  No: 0.6 (95% 0.19–2.14)  Non–Atopic Asthma  No: **3.5 (95%** **1.63–7.53)** |  |
|  |  | Passive smoker |  | Atopic Asthma  No: 1.9 (95% 0.50–6.84)  Non–Atopic Asthma  No: 1.8 (95% 0.88–3.56) |  |
|  |  | Fuel type |  | Atopic Asthma  Natural gas: 3.0 (95% 0.59–14.78)  Non–Atopic Asthma  Natural gas: **4.6 (95%** **1.48–14.46)** |  |
|  |  | Moisture in the home in the last 12 months |  | Atopic Asthma  No: 3.4 (95% 0.92–12.89)  Non–Atopic Asthma  No: 1.5 (95% 0.75–2.81) |  |
| **Karunanayake, 2020** | Asthma in the age group of 12–19 years of FN (health professional diagnosis, ≥6 months duration) | Sex | Odds Ratio | Female: **0.88 (95%** **0.82–0.94)** | |
|  |  | Age (years) |  | <15: 1.00;  ≥15 years: **1.30 (95%** **1.21–1.39)** | |
|  |  | Residence location |  | Urban area: **1.18 (95%** **1.09–1.29)** | |
|  |  | Geographic area |  | Yukon, NWT, & Nunavut: 1.00  Atlantic/Quebec: **2.62 (95%** **2.34–2.93)**  Ontario: **2.46 (95%** **2.19–2.77)**  Manitoba/Saskatchewan: **2.17 (95%** **1.95–2.41)**  Alberta: **3.17 (95%** **2.82–3.57)**  British Columbia: **2.82 (95%** **2.50–3.17)** | |
|  |  | Caregiver’s schooling level |  | Graduate: 1.00  Graduate or no University degree: **1.62 (95%** **1.48–1.77)**;  Some Higher Education or diploma: **1.23 (95%** **1.09–1.38)**;  High School: 0.98 (95% 0.89–1.08)  Some High School or less: **1.22 (95%** **1.08–1.38)** | |
|  |  | Annual income ($) |  | ≥85,000: 1.00  65,000–84,999: 0.96 (95% 0.87–1.07)  45,000–64,999: 0.93 (95% 0.83–1.03)  25,000–44,999: **1.45 (95%** **1.31–1.62)**  <25,000: **1.54 (95%** **1.39–1.70)** | |
|  |  | Smoke in the home |  | Yes: **1.21 (95%** **1.11–1.32)** | |
|  |  | Number of people <18 years in the home |  | None: 1.00;  1–2 people: **1.27 (95%** **1.2–1.44)**;  ≥3 people: 1.13 (95% 0.99–1.30) | |
|  |  | Bronchitis history |  | Yes: **11.89 (95%** **10.23–13.82)** | |
|  |  | BMI |  | Neither obese nor overweight: 1.00;  Overweight: **1.27 (95% 1.16–1.38)**;  Obese: **0.81 (95%** **0.74–0.89)** | |
| **Kinghorn, 2019** | Asthma in the age group of 6–17 years of AI (ICD–9: asthma codes 493.00–493.92, wheeze 786.07; ≥ 2 diagnoses by different providers in 2 years) | **Socioeconomic condition** | | | |
|  |  | Caregiver’s marital status | Odds Ratio | Not married: 1.4 (95% 0.8–2.4) | |
|  |  | Caregiver’s schooling level |  | Below High School: 1.2 (95% 0.5–2.8) | |
|  |  | Household annual income $ |  | <25,000: 1.3 (95% 0.6–2.7) | |
|  |  | Government Insurance Program |  | Yes: 1.2 (95% 0.5–2.5) | |
|  |  | Private insurance plan |  | Yes: 1.3 (95% 0.5–3.1) | |
|  |  | Owned house |  | Yes: 0.9 (95% 0.5–1.8) | |
|  |  | **Home environment characteristics** | | | |
|  |  | Multifamily Housing | Odds Ratio | Yes: 1.4 (95% 0.7–3.0) | |
|  |  | Home occupancy increase |  | >8 occupants: 0.6 (95% 0.3–1.2) | |
|  |  | Insect and rodent infestation |  | 1.6 (95% 0.8–3.2) | |
|  |  | Water damage |  | 1.0 (95% 0.5–1.9) | |
|  |  | Wood–burning stove |  | 2.0 (95% 0.5–7.1) | |
|  |  | Domestic animal |  | 1.0 (95% 0.5–1.9) | |
|  |  | Smoke exposure |  | 0.9 (95% 0.5–1.6) | |
|  |  | **Clinical characteristics of asthma among cases and controls** | | | |
|  |  | Obesity/overweight | Odds Ratio | 1.5 (95% 0.9−2.4) | |
|  |  | Asthma family history |  | **1.9 (95%** **1.1−3.2)** | |
|  |  | Atopy |  | **4.0 (95%** **2.4−6.9)** | |
|  |  | Food allergy |  | **5.9 (95%** **2.2−17.2)** | |
|  |  | RSV infection history |  | **2.9 (95%** **1.6–5.2)** | |
|  |  | Hospitalization |  | **4.4 (95%** **2.3–8.4)** | |
|  |  | Visit to the emergency room of the Indigenous health service |  | **2.6 (95%** **1.4–4.6)** | |
|  |  | Hospitalization in the Indigenous health service |  | 2.4 (95% 0.7–7.9) | |
|  |  | Cough in the last 4 weeks |  | **5.6 (95%** **3.2–10.1)** | |
|  |  | Wheezing in the last 4 weeks |  | **19.0 (95%** **9.1–41.4)** | |
|  |  | Dyspnea in the last 4 weeks |  | **14.1 (95%** **6.9–30.2)** | |
|  |  | School retardment due to illness |  | **6.5 (95%** **2.1–23.6)** | |
| **Best, 2017** | Asthma in the age group of 6–17 years of AI (ICD–9: asthma codes 493.00–493.92, wheeze 786.07, family history V17.5) | BMI (kg/m^2^) | Odds Ratio | **3.21 (95%** **1.20–8.61)** | |
|  |  | >1 high specific antibody |  | **3.88 (95%** **2.37–6.38)** | |
|  |  | Age (years) |  | **0.90 (95%** **0.83–0.99)** | |
|  |  | Genetic variant rs10056340 – 5q22.1 |  | **2.02 (95%** **1.28–3.18)** | |
|  |  | Genetic variant rs2305480 – 17q21 |  | **0.63 (95%** **0.43–0.92)** | |
|  |  | Genetic variant rs7216389 – 17q21 |  | **0.68 (95%** **0.47–0.98)** | |
|  |  | Genetic variant rs8076131 – 17q21 |  | **0.66 (95%** **0.45–0.97)** | |
|  |  | Genetic variant rs4795405 – 17q21 |  | **0.68 (95%** **0.46–0.98)** | |
|  |  | Genetic variant rs9303277 – 17q21 |  | **0.64 (95%** **0.44–0.94)** | |
|  |  | Genetic variant rs6871536 – 5q31 |  | 0.96 (95% 0.59–1.58) | |
|  |  | Genetic variant rs928413 – 9q21 |  | 1.18 (95% 0.63–2.22) | |
|  |  | Genetic variant rs2155219 – 11q13.4 |  | 1.20 (95% 0.84–1.72) | |
|  |  | Genetic variant rs1558641 – 2q11.2 |  | 1.04 (95% 0.45–2.38) | |
| **Best, 2016** | Asthma in the age group of 6–17 years of AI (ICD–9: asthma codes 493.00–493.92, wheeze 786.07, family history V17.5) | Cumulative sensitivity (number of antigens tested with specific IgE above the threshold) |  | **1.75 (95%** **1.33–2.30)** | |
|  |  | Age (years) | Odds Ratio | 0.91 (95% 0.83–1.00) | |
|  |  | BMI (kg/m^2^) |  | 2.47 (95% 0.81–7.58) | |
|  |  | IgE (total, KU/L) |  | 0.99 (95% 0.79–1.26) | |
|  |  | Leukocytes |  | 1.90 (95% 0.67–5.40) | |
|  |  | Eosinophils (%) |  | 1.05 (95% 0.73–1.53) | |
| **Senthilselvan, 2016** | Asthma in the age group of 0–11 years of FN (health professional diagnosis, ≥6 months duration or expected duration) | Age (years) | Odds Ratio | 0–4: 1.00;  5–11: 1.4 (95% 0.81–1.61) | |
|  |  | Sex |  | Female: **0.55 (95%** **0.42–0.72)** | |
|  |  | Allergy |  | Yes: **33.80 (95% 9.03–126.57)** | |
|  |  | Birthweight |  | Normal (≥2500g): 1.00;  Baixo (<2500g): **1.68 (95%** **1.03–2.74)** | |
|  |  | Chronic ear infection |  | Yes: 0.79 (95% 0.45–1.38) | |
|  |  | School attendance |  | Yes: 1.42 (95% 0.98–2.07) | |
|  |  | Physical activity during the week |  | Never: 1.00;  ≤ 1: 1.17 (95% 0.68–1.99);  2–3 times: 1.13 (95% 0.73–1.76);  4–6 times: 0.76 (95% 0.45–1.31);  Every day : **0.65 (95%** **0.42–0.99)** | |
|  |  | Nº children <11 years |  | ≤1: 1.00;  2–3: 0.75 (95% 0.56–1.02);  ≥4: **0.64 (95%** **0.43–0.93)** | |
|  |  | Nº children entre 11–17 years |  | None: 1.00;  1–2: **1.38 (95%** **1.03–1.85)**;  ≥3: 0.59 (95% 0.29–1.22) | |
|  |  | Household income ($) |  | Low (<30.000): 1.00;  Medium (30.000–60.000): 0.84 (95% 0.61–1.16);  High (≥60.000): **1.61 (95%** **1.06–2.46)** | |
|  |  | Smoke–free home |  | Yes: **1.28 (95%** **1.00–1.64)** | |
|  |  | Remote community |  | Isolated–remote/Isolated: 1.00;  Semi–isolated: 0.59 (95% 0.32–1.11);  Non–isolated: 1.31 (95% 0.86–2.01) | |
|  |  | Interaction of allergy with low birthweight |  | No allergy and normal birthweight: 1.00;  Allergy and low birthweight: **6.36 (95%** **1.75–23.07)** | |
| **Overeem, 2014** | Wheezing in the age group of 0–2 years of Amerindian (≥ 2 wheezing episodes in past 12 months, children ≥12 months) | Sex | Odds Ratio | Female: 0.28 (95% 0.06–1.3) | |
|  |  | Developmental delay |  | 4.5 (95% 0.97–21.2) | |
|  |  | Underweight |  | 0.78 (95% 0.15–4.1) | |
|  |  | Helminth infection |  | 0.77 (95% 0.20–3.0) | |
|  |  | Protozoan infection |  | **6.7 (95%** **1.5–30.5)** | |
| **Kraai, 2013** | Asthma in the age group of 2–10 years of Warao (ISAAC definition – self reported wheeze in last 12 months) | Age (years) | Odds Ratio | 2–3: 1.00;  4–5 years: **0.51 (95%** **0.30–0.86)**;  6–7 years: 0.68 (95% 0.40–1.14);  8–10 years: **0.46 (95%** **0.27–0.78)** | |
|  |  | Sex |  | Female: 0.74 (95% 0.50–1.08) | |
|  |  | Geographic area |  | Curiapo: 1.00;  Manuel Renald: 0.67 (95% 0.36–1.26);  Padre Barral: 0.61 (95% 0.37–1.01);  Santos de Abalgas: 0.52 (95% 0.25–1.07) | |
|  |  | Cooking method |  | Gas and wood: 1.44 (95% 0.78–2.67);  Wood: **2.12 (95%** **1.18–3.84)** | |
|  |  | House (walls) |  | With walls: 1.00;  Without walls: **1.83 (95%** **1.20–2.79)** | |
|  |  | Number of cigarettes smoked per day in the household |  | 1–10 cigarettes/day: 0,89 (95% 0.57–1.38);  >10 cigarettes/day: **2.69 (95%** **1.11–6.48)** | |
| **Chang, 2012** | Asthma in the age group of 6–14 years of NAI (physician/nurse/health professional diagnosis; attack in past 12 months) |  | Odds Ratio |  | |
|  |  | Sex |  | Male: **1.52 (95%** **1.33–1.74)** | |
|  |  | Aborigine ancestral |  | North American Indian: 1.00;  Innuits: **0.61 (95%** **0.43–0.87)**;  Metis: 1.13 (95% 0.97–1.32);  Multiple Ancestors: 1.07 (95% 0.91–1.26) | |
|  |  | Residence location |  | Urban: 1.00;  Non–urban (rural): **0.82 (95%** **0.72–0.94)** | |
|  |  | Geographic area |  | Prairies: 1.00;  Atlantic: 1.12 (95% 0.93–1.35);  Quebec: 1.12 (95% 0.95–1.34);  Ontario: 1.00 (95% 0.85–1.17);  British Columbia: 0.89 (95% 0.75–1.05);  Territories: **0.36 (95%** **0.28–0.48)** | |
|  |  | Low birthweight <2500g |  | Yes: **1.44 (95%** **1.15–1.80)** | |
|  |  | Already breastfed |  | Yes: 0.97 (95% 0.86–1.10) | |
|  |  | Daycare attendance |  | Yes: 1.13 (95% 0.99–1.29) | |
|  |  | BMI |  | Normal/underweight: 1.00;  Overweight: **1.17 (95%** **1.00–1.38)**;  Obese: **1.24 (95% 1.05–1.48)** | |
|  |  | Allergies |  | Yes: **5.99 (95%** **5.26–6.84)** | |
|  |  | Health appointments in the last 12 months |  | Yes: **2.03 (95%** **1.74–2.38)** | |
|  |  | Hospitalization in the last 12 months |  | Yes: **1.78 (95%** **1.35–2.34)** | |
|  |  | General health status |  | Excellent or very good: 1.00;  Good, fair, or poor: **2.92 (95%** **2.59–3.30)** | |
|  |  | Neurological/psychological difficulties |  | Yes: 1.27 (95% 0.97–1.66) | |
|  |  | Housing conditions |  | Not requiring repair: 1.00;  Requiring minimum repair: 1.08 (95% 0.93–1.24);  Requiring significant repair: **1.27 (95%** **1.06–1.54)** | |
|  |  | Household income/year $ |  | >30,000: 1.00;  ≤ 30,000: 1.16 (95% 0.99–1.35) | |
| **Ye, 2012** | Asthma in the age group de 0–6 years of AI (positive response to having asthma and physician/nurse/health professional diagnosis) | Age (years) | Odds Ratio | 0–1 year: 1.00;  2–3 years: **2.10 (95%** **1.71–2.58)**;  4–6 years: **2.53 (95%** **2.06–3.12)** | |
|  |  | Sex |  | Male: **1.67 (1.43–1.95)** | |
|  |  | Ancestry |  | North American Indian: 1.00;  Metis: 0.87 (95% 0.73–1.05);  **Innuits: 0.64 (95% 0.47–0.86);**  Multiple Ancestors: 0.97 (95% 0.79–1.19) | |
|  |  | Number of older siblings |  | 0: 1.00;  1: 1.13 (95% 0.96–1.34);  ≥2: **1.26 (95%** **1.04–1.54)** | |
|  |  | Housing type |  | House: 1.00;  Apartment or asset: **1.47 (95%** **1.23–1.76)** | |
|  |  | Healthcare access |  | Limited: 1.00;  Easy: **0.42 (95%** **0.34–0.52)** | |
|  |  | Birthweight |  | ≥2500g: 1.00;  ≤2500g: **1.70 (95%** **1.32–2.20)** | |
|  |  | Breastfeeding |  | No: 1.00**;**  Always, but not exclusive: 0.84 (95% 0.70–1.01);  Exclusive: **0.59 (95%** **0.44–0.78)** | |
|  |  | Attends daycare |  | Yes: **1.32 (95%** **1.13–1.55)** | |
|  |  | Ear infection |  | Yes**: 2.17 (95%** **1.82–2.58)** | |
| **Shepherd, 2012** | Asthma in the age group de 0–17 years of Aborigines (parental report) | Schooling (main caregiver) | Odds Ratio | No schooling: 0.60 (95% 0.33–1.36);  Nine schooling years or less: 0.75 (95% 0.64–1.04);  10 schooling years: 1.00;  11–12 schooling years: 1.08 (95% 0.84–1.36);  13 schooling years and above: 1.33 (95% 0.89–1.86) | |
|  |  | Schooling (secondary caregiver): |  | No schooling: 0.89 (95% 0.48–1.57);  Nine schooling years or less: 0.90 (95% 0.67–1.19);  10 schooling years: 1.00;  11–12 schooling years: 1.31 (95% 0.88–1.69);  13 schooling years and above: 0.85 (95% 0.44–1.88) | |
|  |  | No secondary caregiver |  | 1.22 (95% 0.90–1.45) | |
|  |  | Occupation |  | Worker, administrative staff, and merchant: 1.00;  Managers/professionals: 1.08 (95% 0.70–1.67);  Unemployed: 1.01 (95% 0.79–1.30) | |
|  |  | Family financial strain |  | Spend more than they receive: 0.95 (95% 0.49–1.84);  Just enough to survive: 0.88 (95% 0.47–1.62);  Some money was saved, but they spent it: 0.78 (95% 0.41–1.50);  They could save some money: 0.96 (95% 0.53–1.76);  They could save much money: 1.00 | |
|  |  | Housing ownership |  | Owned: 1.00;  Being paid: **1.97 (95%** **1.00–3.88)**;  Rented: **1.90 (95%** **1.02–3.53)**;  Other: 0.66 (95% 0.23–1.91) | |
|  |  | Number of poor housing quality indicators |  | None: 1.00;  1: 0.99 (95% 0.76–1.30);  2: 0.94 (95% 0.68–1.29);  ≥3: **0.60 (95% 0.43–0.85)** | |
|  |  | Socioeconomic index quintiles by area (SEIFA) |  | Second: 1.48 (95% 1.10–2.00);  Third: 1.80 (95% 1.29–2.51);  Fourth: 1.41 (95% 0.83–2.37);  Upper quintile: **3.48 (95% 1.34–9.04)** | |
|  |  | Quintiles of the index referring to Indigenous socioeconomic results (IRISEO) |  | Second: **3.37 (95%** **2.07–5.49)**;  Third: **3.91 (95%** **2.42–6.31)**;  Fourth: **4.66 (95%** **2.80–7.74)**;  Upper quintile: **9.24 (95%** **3.10–27.20)** | |
| **Crighton, 2010** | Asthma in the age group of 0–14 years in FN (physician/nurse/health professional diagnosis; attack in past 12 months/ regular asthma medication use) | Age (years) | Odds Ratio | 0–14 years: **1.03 (95%** **1.01–1.05)** | |
|  |  | Sex |  | Male: **1.63 (95%** **1.34–1.99)** | |
|  |  | Ethnicity |  | Metis 0.93 (95% 0.73–1.17);  **Innuits 0.70 (95% 0.46–1.05)**;  Other/Mixed 0.79 (95% 0.59–1.07) | |
|  |  | Domicile outside the reserve (urban) |  | **1.25 (95%** **1.04–1.50)** | |
|  |  | Domicile in rural area |  | 1.00 (95% 0.80–1.27) | |
|  |  | Regions |  | Atlantic: 1.00;  Quebec – 0.67 (95% 0.41 – 1.10);  Ontario – 1.18 (95% 0.80–1.73);  Prairies – 0.74 (95% 0.53–1.02);  British Columbia: **0.67 (95%** **0.47–0.95)**;  Northern Territories: **0.38 (95%** **0.26–0.56)**; | |
|  |  | Schooling |  | High School or more: 1.00;  Below High School: 0.95 (95% 0.77–1.18) | |
|  |  | Homes in need of major repairs |  | **1.31 (95%** **1.01–1.70)** | |
|  |  | House construction year |  | <1961: 1.16 (95% 0.87–1.56);  1961–1980: 0.97 (95% 0.78–1.20);  >1980: 1.00 | |
|  |  | Income ($CAD) |  | <$20,000: 1.20 (95% 0.90–1.60);  $20,000–<$40,000: 1.21 (95% 0.93–1.57);  $40,000–$60,000: 0.88 (95% 0.66–1.18);  >$60,000: 1.00 | |
|  |  | Recent visit to health services |  | **1.01 (95% 1.00–1.02)** | |
| **Surdu, 2006** | Asthma in Mohicans aged 2–14 years (definition not specified) | Asthma family history | Odds Ratio | 1.37 (90% 0.15–12.81) | |
|  |  | Smoke in the house in the last 12 months |  | 1.49 (90% 0.52–4.23) | |
|  |  | Smoke in the house during the child’s life |  | 1.18 (90% 0.45–3.09) | |
|  |  | Garage attached to the house |  | 1.31 (90% 0.39–4.43) | |
|  |  | Burning barrel near home (5min walk) |  | 1.56 (90% 0.52–4.74) | |
|  |  | Pets at home |  | 0.73 (90% 0.29–1.85) | |
|  |  | Moisture in walls, ceilings, carpets, and furniture |  | 0.95 (90% 0.30–3.05) | |
|  |  | Mold in the house |  | 0.83 (90% 0.30–2.29) | |
|  |  | Cockroach, ant, and other insects in the house |  | 0.77 (90% 0.30–1.99) | |
|  |  | Smoking during pregnancy |  | 1.26 (90% 0.41–3.90) | |
|  |  | Preterm birth |  | 2.12 (90% 0.67–6.69) | |
|  |  | Breastfeeding in childhood |  | **0.18 (90% 0.05–0.75)** | |
|  |  | Daycare in the first two years of life |  | 0.53 (90% 0.18–1.57) | |
| **Lewis, 2004** | Asthma in AI/NA in the age group of 10–18 years (ISAAC questionnaire) | **Asthma** | | | |
|  |  | Sex | Relative Risk | Female: 1.15 (95% 0.48–2.73) | |
|  |  | Age (years) |  | 0.94 (95% 0.67–1.32) | |
|  |  | House in the village (rural) |  | 0.43 (95% 0.16–1.12) | |
|  |  | Exposure to cigarette smoke |  | High: **3.90 (95%** **1.38–11.00)** | |
|  |  | Active smoker |  | 0.60 (95% 0.17–2.16) | |
|  |  | Unknown tobacco use status |  | 0.67 (95% 0.21–2.15) | |
|  |  | Potential atopy |  | 1.37 (95% 0.36–5.17) | |
|  |  | **Asthma–like** | | | |
|  |  | Sex | Relative Risk | Female: 1.70 (95% 0.80–3.63) | |
|  |  | Age (years) |  | 1.10 (95% 0.86–1.41) | |
|  |  | House in the village (rural) |  | **0.37 (95%** **0.17–0.79)** | |
|  |  | Exposure to cigarette smoke |  | High: **2.76 (95%** **1.37–4.70)** | |
|  |  | Active smoker |  | **3.17 (95%** **1.50–5.39)** | |
|  |  | Unknown tobacco use status |  | **2.87 (95% 1.36–4.99)** | |
|  |  | Potential atopy |  | **3.86 (95%** **1.78–5.32)** | |
| **Schei, 2004** | Asthma Guatemalan Indigenous people in the age group of 4–6 years (ISAAC questionnaire) | Always wheezing | Odds Ratio | **2.0 (95%** **1.1–3.7)** | |
|  |  | Wheezing in the last 12 months |  | **3.4 (95% 1.3–8.5)** | |
|  |  | Diagnosed asthma |  | 1.8 (95% 0.76–4.19) | |
|  |  | Exercise–induced wheezing in the past year |  | **3.5 (95%** **1.4–8.6)** | |
|  |  | Nighttime cough in the last year |  | 1.00 | |
|  |  | Speech limit to 1–2 words |  | **3.4 (95%** **1.1–11.3)** | |
|  |  | Wheezing episodes in the last year |  | 1–3 episodes: 2.0 (95% 0.54–0.77);  >12 episodes: 2.7 (95% 0.83–10.80) | |
|  |  | Waking up more than once a week in the last year |  | 1.8 (95% 0.52–5.44) | |
| **Clark, 1995^*^** | Asthma of NAI in the age group of 3–13 years (parent–reported physician diagnosis) and/or wheeze (acute: attack causing shortness of breath + ≥ 2 episodes or medication; persistent: wheezing apart from colds) | Sex (male) | Odds Ratio | Male: **2.2 (95%** **1.0–4.9)** | |
|  |  | Bronchiolitis (Yes) |  | Yes: **19.4 (95%** **5.5–76.3)** | |
|  |  | Breastfeeding (Yes) |  | Yes: 0.6 (95% 0.2–1.3) | |
|  |  | Passive exposure to cigarette smoke from a parent |  | Children <2 years: 1.4 (95%0 .5–3.8);  Children >2 years: 1.4 (95% 0.5–3.5);  Regularly: 0.4 (95% 0.01–2.5) | |
|  |  | Home heating |  | Electric heater: **0.2 (95%** **0.02–0.8)**;  Wall oven: 0.5 (95% 0.1–1.5);  Fireplace: 0.5 (0.2–1.01) | |
|  |  | Wood–burning stove |  | 0.8 (95% 0.3–1.9) | |

OR, odds ratio; RR, relative risk; CI, confidence interval; FN, first nation; NAI, native American Indian; AI, American Indian; AN, Alaskan Native; NWT, Northwestern Territories and BMI, body mass index. * *Data are unadjusted and from univariate analysis.
